# Vinegar Metabolomics: An Explorative Study of Commercial Balsamic Vinegars Using Gas Chromatography-Mass Spectrometry

**DOI:** 10.3390/metabo6030022

**Published:** 2016-07-23

**Authors:** Farhana R. Pinu, Samuel de Carvalho-Silva, Ana Paula Trovatti Uetanabaro, Silas G. Villas-Boas

**Affiliations:** 1 The New Zealand Institute for Plant & Food Research Limited, Private Bag 92169, Auckland 1142, New Zealand; 2 Department of Biological Sciences, State University of Feira de Santana, Feira de Santana 44036-900, Brazil; samuelmicrobiologia@gmail.com; 3 CAPES Foundation, Ministry of Education of Brazil, Brasília DF 70040-020, Brazil; 4 Agro-industry Microbiology Laboratory, Department of Biological Sciences, State University of Santa Cruz, Ilhéus 45662-900, Brazil; aptuetanabaro@gmail.com; 5 School of Biological Sciences, University of Auckland, Private Bag 92019, Auckland 1010, New Zealand; s.villas-boas@auckland.ac.nz

**Keywords:** metabolomics, metabolite profiling, amino acids, organic acids, fatty acids, aroma compounds, geographic indication

## Abstract

Balsamic vinegar is a popular food condiment produced from cooked grape must by two successive fermentation (anaerobic and aerobic) processes. Although many studies have been performed to determine the composition of major metabolites, including sugars and aroma compounds, no study has been undertaken yet to characterize the comprehensive metabolite composition of balsamic vinegars. Here, we present the first metabolomics study of commercial balsamic vinegars by gas chromatography coupled to mass spectrometry (GC-MS). The combination of three GC-MS methods allowed us to detect >1500 features in vinegar samples, of which 123 metabolites were accurately identified, including 25 amino acids, 26 carboxylic acids, 13 sugars and sugar alcohols, four fatty acids, one vitamin, one tripeptide and over 47 aroma compounds. Moreover, we identified for the first time in vinegar five volatile metabolites: acetin, 2-methylpyrazine, 2-acetyl-1-pyroline, 4-anisidine and 1,3-diacetoxypropane. Therefore, we demonstrated the capability of metabolomics for detecting and identifying large number of metabolites and some of them could be used to distinguish vinegar samples based on their origin and potentially quality.

## 1. Introduction

The history of vinegar production coincides with that of winemaking, which has been documented over 5000 years ago. Since then, vinegar has been used as a condiment, in the preservation of food, and as a household cleaning agent. Moreover, vinegar is known for its antioxidant properties and health benefits, including the prevention of inflammation and hypertension [1,2]. It has also been reported that regular intake of vinegar decreases serum cholesterol, triacylglycerol and blood glucose concentrations [3,4]. Recently, vinegar has gained popularity because of its potential use in weight loss [5]. Regular vinegar intake also can improve ovulary function in women with polycystic ovarian syndrome [6]. Although vinegar has many applications in our everyday lives, economically it has always been considered non-profitable. However, balsamic vinegar has high economic value compared with other vinegars. Therefore, there is a growing scientific and commercial interest in balsamic vinegars and in better characterizing their fine composition [7,8,9,10].

Balsamic vinegars are usually produced from the juice of white grapes (e.g., Lambrusco, Trebbiano and Spergola) that are cooked to concentrate by 50% before placing them in the series of wooden casks called “batteria” where they undergo both alcoholic and acetic fermentation. The resulting vinegar is then matured in a smaller wooden barrel for many years (10–25 years) to achieve the expected viscosity, sweetness, intensity, flavor and aroma profile [11]. These traditional balsamic vinegars (TBV) are the specialty of the Modena and Reggio-Emilia regions of Italy and there are strong regulations to maintain the product quality of these TBV. However, the commercial balsamic vinegars are produced in slightly different ways and the prices also differ from that of TVB because of the difference in quality. Many studies have been carried out to determine the profiles of a few targeted compounds, including volatile and polyphenolic compounds, in both TVB and other commercial balsamic vinegars [12,13,14,15,16,17,18,19,20]. The effect of aging in woods has been studied widely, while special attention has been paid to the originality and authentication of TVB [7,12,13,14]. However, only a few studies have been conducted to determine the fine composition of balsamic vinegars [21,22,23,24]. To the best of our knowledge, no study has been undertaken yet to determine the comprehensive metabolite profiles that would allow the detection and identification of all major metabolite groups (amino acids, carboxylic acids, fatty acid, volatile compounds, and sugar and sugar alcohols), including potential contaminants present in vinegars.

Recent advancements in analytical techniques have made it possible to determine many groups of metabolites present in biological samples. However, the proper choice of instrumental technique is very important in metabolome analysis. GC-MS and nuclear magnetic resonance (NMR) spectroscopy have been widely used for the analysis of different vinegars [13,14,21]. Both analytical instruments have their own merits and demerits. GC-MS is one of the most mature technologies in metabolomics; it is very sensitive and allows the simultaneous analysis of hundreds of metabolites [25,26]. However, one limitation is that the metabolites have to be volatile so that they can be separated in a gas phase. Therefore, chemical derivatization is often required to make the analytes more volatile and stable at high temperatures [27]. Nevertheless, the major advantage of using GC-MS is that the identification of metabolites is comparatively easier than with any other existing techniques because of the availability of many in-house and commercial interchangeable MS libraries [28]. By contrast, NMR is less destructive and can provide accurate identification of a molecule. However, data interpretation can be complicated and it has a relatively low sensitivity that renders NMR inappropriate for profiling hundreds of compounds simultaneously. Moreover, GC-MS is known for providing a far better comprehensive metabolite profile of food products and beverages than Nuclear Magnetic Resonance (NMR) analysis [29,30]. Therefore, our instrument of choice for this study was GC-MS.

This is an explorative study where one of our aims was to optimize GC-MS methods for global metabolite profiling of commercial balsamic vinegars, to identify as many groups of metabolites as possible. Moreover, here we also show how the application of metabolomics allows us to distinguish commercial balsamic vinegars by determining the key metabolites that play significant roles in vinegar originality and perhaps quality.

## 2. Results and Discussion

### 2.1. Differentiation between the Vinegar Samples Based on Their Comprehensive Metabolite Profiles

In this study, we successfully optimized three previously published GC-MS methods [27,30,31] and all these methods showed excellent reproducibility and linearity for the analysis of commercial balsamic vinegars. The residual standard deviation (RSD) was below 20% for most of the identified metabolites (Table 1 and Table 2), which confirms that our data were of good quality [31,32]. We were able to detect over 1500 features in the balsamic vinegar samples, among which 120 metabolites were positively identified using in-house and commercial MS libraries, which contain information about retention time and mass fragments of metabolites. Among these metabolites, there were least 25 amino acids, 26 carboxylic acids, 13 sugars and sugar alcohols, four fatty acids, one vitamin, one tripeptide and over 47 aroma compounds including esters, higher alcohols, volatile organic acids, aldehydes and ketones (Table 1 and Table 2). Therefore, the combination of three different GC-MS methods indeed allowed us to determine a wide range of metabolites present in the commercial balsamic vinegar samples.

The concentration of metabolites in balsamic vinegars depends on at least several factors, including the raw materials, strains of microorganisms, overall production and aging process [33]. Among 73 non-volatile metabolites detected and identified in this study, only 14 of them showed significant difference in six different balsamic vinegars (*p* < 0.05). We found a production area specific difference in amino acid concentrations among the balsamic vinegars. For instance, vinegars M1 and M4 (both bottled at Pertini 440-41032, Cavezzo) contained lower concentrations of threonine and aspartic acid, whilst P and D (both bottled at n°CSQA 216311) presented comparatively higher concentrations of these proteogenic amino acids (Table 1). Interestingly, the concentrations of glutathione were higher in IGP vinegars, while non-IGP vinegars contained more aspartic and glutamic acid (Table 1). This observation indicates that non-IGP vinegars were less mature (green characteristics) than the IGP vinegars as they contained more acidic metabolites. We also found that the levels of malic, succinic, tartaric, lactic, citramalic and glyceric acids are much lower in IGP vinegars than in non-IGP vinegars (Table 1), which again confirms the green characteristics of the non-IGP vinegars. Our data also shows that concentrations of mannitol and galactose were comparatively higher in non-IGP balsamic vinegars than in the IGP vinegars (Table 1). Thus, similarly to organic acids, mannitol and galactose also could be used as potential markers for determining the origin of balsamic vinegars and perhaps even quality.

Aroma compounds are a major group of metabolites that have a direct contribution to the sensory properties and overall quality of any type of food. This group of metabolites in vinegar develops during different stages of the production process [13,34,35,36,37]. The types of grape juice, fermentation conditions, microorganisms used during the production, the aging condition and materials have a huge impact on the development of volatile metabolites [9,34,35,36,37]. Table 2 shows the relative abundance of over 50 aroma compounds present in the balsamic vinegar samples. Acetic acid is obviously the main volatile metabolite in vinegar (present in g/L and the amount of this organic acid was similar in all the samples. We have found that the concentrations of butyric acid, furoic acid, propanoic acid, diethyl succinate, isoamyl acetate, phenylethyl acetate, 2-methyl-1-butanol, isoamyl alcohol and methionol were higher in IGP vinegars, whilst non-IGP vinegar samples contained high amounts of benezeneacetic acid, 2-methylpyrazine, 4-anisidine and 2-acetyl pyrolline (Table 2). Among them, only isoamyl alcohol and isoamyl acetate (which develop during fermentation) are known to be present at microgram per liter concentrations [38]. However, our data made it clear that all these volatile metabolites present in minor concentrations could also be used to differentiate different balsamic vinegar samples. Although less concentrated metabolites were mostly disregarded in most of the studies carried out on balsamic vinegar, it is now time to pay more attention on them as these metabolites also might have significant roles in sensory properties.

**Figure 1:**
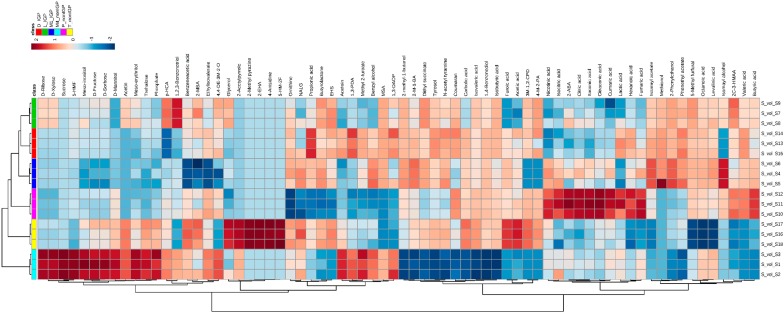
Hierarchical cluster analysis performed using metabolite profiles of balsamic vinegars. The heatmap was generated using 70 most significant metabolites (*p*-value < 0.05) by a Ward algorithm and Euclidean distance analysis. IGP denotes the samples certified as Protected Geographic Indication to indicate their premium quality, while non-IGP samples are regular commercial vinegar samples. Here, D = Delmaine^TM^, L = Lupi^TM^, M1 = Mazetti^TM^ one leaf, M4 = Mazetti^TM^ four leaves, P = Pam’s^TM^ and T = Tastemaker^TM^, 5-HMF = 5-Hydroxymethylfurfural, *p*-HCA = *p*-hydroxycinnamic acid, 2-MBA = 2-methylbutyric acid, 4,4-DE-3M-2-O = 4,4-Diethyl-3-methylene-2-oxetanone, 2-EHA = 2-Ethylhexanoic acid, 5-HM-2-F = 5-Hydroxymethyl-2-furaldehyde, NALG = *N*-Acetyl-glutamate, EHS = Ethylhydrogen succinate, 1,3-PDA = 1.3-Propylene diacetate, MSA=Methylsuccinic anhydride, 1,3-DAOP = 1,3-Diacetoxypropane, 2-M-1-BA = 2-Methyl-1-butyl acetate, 3-M-1,2-CPD = 3-Methyl-1,2-cyclopentanedione, 4-M-2-PDA = 4-Methyl-2-pentanoic acid, 2-ABA = 2-Aminobutyric acid.

We performed a hierarchical cluster analysis (HCA) using 70 statistically significant metabolites (*p*-value < 0.05) identified by three GC-MS methods (Figure 1), which clearly shows that each vinegar has a distinct metabolite profile, as expected. In addition, Figure 1 also shows that HCA based on comprehensive metabolite profiles clearly distinguished between two types of vinegars analyzed in this study: vinegar samples certified as Protected Geographic Indication (IGP) and non-IGP certified vinegars. Especially two non-IGP vinegars, M4 and T, formed a completely separate cluster, while the other one (P) formed a separate brunch closer to the IGP vinegars mainly because of their similarity in sugar composition. The separation between P and other two non-IGP vinegars can also be explained by the difference between their production areas, which clearly affected their overall metabolite composition (Table 1). However, we noted that some of the vinegars (e.g., M1 and M4; and P and D) clustered slightly apart from each other despite being produced in the same geographical location. But some (M1 and D) had IGP certification and others (P and M4) did not. “Protected denomination” is a legislative system in the European Union that provides recognition for products from certain geographical areas based on their production system and quality [13]. IGP is generally a regional certification for the balsamic vinegars and the producers usually need to maintain premium quality in terms of sensory-chemical features as well as production process in order to achieve this certification [9,13,18,24]. Therefore, we expected to see the differences between IGP and non-IGP vinegars, as their production processes are very different. Taken all the data together, we assume that organic acids could be good indicators for the determination of balsamic vinegar quality and they might also have a negative effect on the sensory quality of vinegars when their concentrations increase beyond a certain threshold [9,34]. Inexpensive and somewhat inferior vinegars usually contain considerably high amount of total acids [9,34] as the producers tend to add different organic acids (e.g., acetic and citric acids) to increase the acidity of their product. It is also noteworthy that the fermentation and maturation time for IGP vinegars are comparatively longer (at least 90 days of maturation) [13,18] than other commercial vinegars. Thus, once again, the presence of higher amount of organic acids can be simply related to the fact that non-IGP vinegars are less mature.

### 2.2. Five Volatile Metabolites Reported De Novo in Balsamic Vinegars

**Figure 2:**
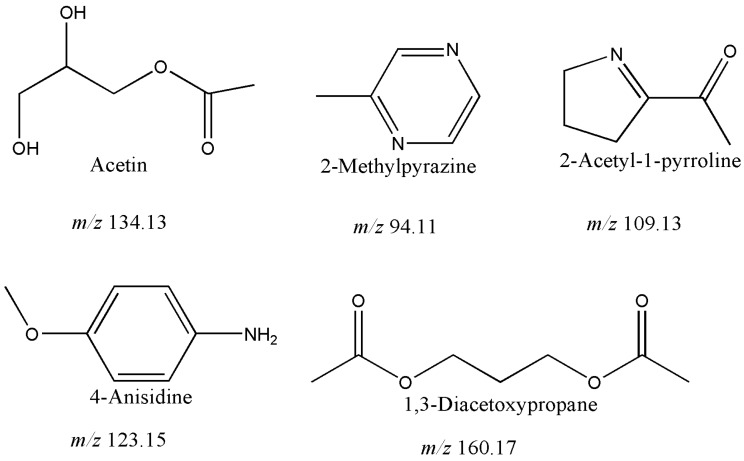
First identification of five volatile metabolites in balsamic vinegar samples showing their chemical structures and molecular weights.

One of the most interesting findings from our study is that we detected five metabolites that have been never associated with vinegar before. Figure 2 shows the structures and molecular weights of acetin, 2-methylpyrazine, 2-acetylpyrolle, 4-anisidine and 1,3-diacetoxyporpane found in the balsamic vinegars analyzed in this study. It is still not clear how these metabolites develop during the production process. For instance, acetin might be a contaminant from the production instruments, as it is widely used as a fuel additive [39]. Similarly, 1,3-diacetoxyporpane (also known as 1,3-propanediol-diacetate) is also used in combustion engines [40]; therefore, it also might be an equipment-derived contaminant. 2-methylpyrazine (nutty flavor) and 2-aetyl-1-pyrolline (rice flavor) on the other hand are well-known food flavors [41,42] and these were present only in non-IGP vinegars, thus might be derived from the flavor enhancing agents used during the production. Therefore, it seems that these metabolites either could be potential contaminants or food additives. Contaminants and pollutants can be introduced in the different stages of balsamic vinegar production and we showed that metabolomics could be an excellent approach to detect and identify this type of metabolites.

## 3. Materials and Methods

### 3.1. Chemicals

All the chemicals used for this study were of analytical grade. Internal standards and reagents for GC-MS, such as l-alanine-2,3,3,3-*d*_4_ (*d*_4_-alanine), d-ribitol, 12-bromo-1-dodecanol, *N*-methyl-*N* (trimethylsilyl)trifluoroamide (MSTFA), pyridine, diethyl ether, and all other metabolite standards, were purchased from Sigma-Aldrich (St. Louis, MO, USA). Methoxyamine hydrochloride was obtained from Fluka (Steinheim, Switzerland), anhydrous sodium sulfate from BDH chemicals (Poole, UK), and acetone from Biolab (Scoresby, Australia). Methanol, sodium hydroxide and sodium bicarbonate were purchased from Merck (Darmstadt, Germany). All the chemical solutions were prepared using Grade 1 water (BARNSTEAD^®^ NANOpure Diamond™ Water Purification System, Waltham, MA, USA) or absolute ethanol (UNIVAR, AJAX FINECHEM, Auckland, New Zealand).

### 3.2. Vinegar Samples

Six commercial balsamic vinegars were purchased from two different supermarkets in Auckland, New Zealand. Among them, five were produced in Modena, Italy, and one was produced and packed in New Zealand from balsamic vinegar exported from Modena (Table 3). The acidity of all these vinegar samples was 6%, as reported by the producers. Only three of the vinegar samples were certified as Protected Geographic Indication (IGP), thus indicating that they have been produced under strict regulations in the regions of Modena and Reggio Emilia, Italy (Table 3).

### 3.3. Metabolite Profiling of Balsamic Vinegars by GC-MS

All the balsamic vinegar samples were analyzed in triplicate using three different GC-MS methods.

#### 3.3.1. Methylchloroformate (MCF) Derivatization

MCF derivatization of balsamic vinegars was carried out to determine the profile of amino and non-amino organic acids, and some primary amines and alcohols. The sample preparation protocol was adopted from that of Pinu et al. [30] and then optimized for the analysis of vinegars. An amount of 130 µL of vinegar was mixed with 20 µL internal standard l-alanine-2,3,3,3-*d*_4_ (10 mM) and 50 µL of NaOH (2 M) in silanized reaction tubes. MCF derivatization was performed according to the method of Smart et al. [27] After derivatization, all the samples were injected into an Agilent GC 7890 coupled to an Agilent MSD 5975 with a quadrupole mass selective detector (Electron Ionization; positive mode) operated at 70 eV. The GC column used for all analyses was a Zebron ZB-1701 (Phenomenex, Torrance, CA, USA), 30 m × 250 µm (internal diameter) × 0.15 µm (film thickness), with a 5-m guard column. The MS was operated in scan mode (start after 6 min; mass range 38–650 a.m.u. at 1.47 scans/s). All the other analytical parameters are described in Smart et al. [27].

#### 3.3.2. Trimethyl Silyl (TMS) Derivatization

The trimethylsilyl (TMS) derivatization method was used for the analysis of sugar, sugar alcohols, amino sugars and their derivatives. Balsamic vinegar samples were diluted 100 times and then 20 µL of diluted samples were mixed with 60 µL of methanol and 20 µL of d-ribitol (internal standard) (adopted from the method of Pinu et al. [30]). A rotary vacuum dryer (Thermo Fischer, Holbrock, NY, USA) was used to dry the samples completely. The TMS derivatization was performed following the protocol published in Villas-Bôas et al. [43] and the derivatized vinegar samples were injected into an Agilent GC 7890 coupled to a MSD 5975 (Agilent Technologies, St Loius, MA, USA) with a quadrupole mass selective detector (Electron Ionization; positive mode) operated at 70 eV. The column used for the analysis of TMS-derivatized samples was a Zebron ZB-1701 (Phenomenex), 30 m × 250 µm (internal diameter) × 0.15 µm (film thickness), with a 5-m guard column. The MS was operated in scan mode, where scanning started after 5 min (mass range 40 to 650 a.m.u at 1.47 scans/s).

#### 3.3.3. Analysis of Volatile Metabolites

The sample preparation protocol for the analysis of volatile compounds in balsamic vinegars was also adopted from that of Pinu et al. [30]. In this method, 2 mL of vinegar samples were mixed with 1 mL of diethyl ether in a 10-mL screw-capped glass test tube. Internal standard 12-bromo-1-dodecanol (10 mM, 5 µL) was added to the samples. The test tube was capped tightly to avoid loss of any volatile compounds. The sample was vigorously mixed for 2 min using a vortex mixer. Then the samples were centrifuged for 2 min at 2000 rpm to separate the polar and non-polar layers. About 500 µL of the diethyl ether layer was transferred to a silanized glass tube and concentrated to about 50 µL using N_2_ gas. The concentrated samples were transferred to a GC-vial and analyzed by an Agilent GC 7890 coupled to a MSD 5975 (Agilent Technologies) with a quadrupole mass selective detector (Electron Ionization; positive mode) operated at 70 eV using the same column described for analysis of MCF and TMS derivatives. All other parameters of this method are described in Pinu et al. [30].

#### 3.3.4. Data Mining and Statistical Analyses

GC-MS data mining was carried out according to the methods of Aggio et al. [44] and Pinu et al. [30] using in-house R-based software and scripts (Version 3.0.1). ANOVA and t-tests were also performed using two different in-house R scripts. For other data analysis, Microsoft^®^ Excel 2007 and SigmaPlot 12.0 were used. Hierarchical Cluster Analysis (HCA) was performed using a web interface, Metaboanalyst 3.0 (http://www.metaboanalyst.ca), created by the University of Alberta, Canada [45].

## 4. Conclusions

Our study was explorative in nature and a proof of concept that shows the potential of comprehensive metabolomics approach to distinguish different balsamic vinegars. Here, we successfully combined the data from three different GC-MS methods, which allowed us to detect and identify over 120 metabolites in commercial balsamic vinegar samples. Our results clearly indicated that we were able to distinguish different balsamic vinegars based on their metabolite profiles. Although most of the published studies on balsamic vinegars have measured only a few major metabolites (e.g., glucose, acetic acid, and furan compounds), this study has demonstrated that many metabolites in balsamic vinegars that were considered minor could be used as markers of originality or perhaps quality.

## Figures and Tables

**Table 1:** Features and levels of non-volatile metabolites detected and identified in balsamic vinegars.

Metabolite	RT (min)	*m*/*z*	Avg RSD (%); *n* = 6	Relative Abundance in Vinegar Samples					
				D	M1	L	T	M4	P
**Amino acids (25):**									
2-aminobutyric acid	11.40	116	10	0.08 ± 0.01	0.10 ± 0.00	0.11 ± 0.00	0.08 ± 0.01	0.10 ± 0.02	0.28 ± 0.05
4-aminobutyric acid (GABA)	14.98	102	11	0.62 ± 0.09	0.69 ± 0.05	0.47 ± 0.09	0.35 ± 0.05	0.46 ± 0.12	0.41 ± 0.06
4-Hydroxyproline	12.75	144	9	ND	0.08 ± 0.00	0.04 ± 0.00	ND	0.07 ± 0.01	0.12 ± 0.03
Alanine	11.15	102	6	2.77 ± 0.55	3.05 ± 0.16	2.08 ± 0.52	1.89 ± 0.61	2.81 ± 0.24	3.87 ± 0.59
Asparagine	16.70	70	11	0.17 ± 0.02	0.23 ± 0.04	ND	ND	0.16 ± 0.09	0.20 ± 0.04
*Aspartic acid*	*16.45*	*160*	*11*	*0.51 ± 0.08*	*0.54 ± 0.03*	*0.42 ±0.09*	*0.82 ±0.03*	*0.72 ±0.12*	*0.60 ±0.08*
Beta-Alanine	12.75	88	7	0.05 ± 0.00	0.06 ± 0.00	0.04 ± 0.00	0.08 ± 0.09	0.06 ± 0.02	0.04 ± 0.00
Glycine	*11.50*	*88*	*11*	*0.52* ± 0.12	*0.49* ± 0.18	*0.34* ± 0.04	*0.42* ± 0.16	*0.44* ± 0.06	*0.98* ± 0.17
*Glutamic acid*	18.20	174	14	0.15 ± 0.05	0.14 ± 0.00	0.21 ± 0.06	0.44 ± 0.12	0.40 ± 0.08	0.57 ± 0.03
Glutamine	17.99	174	24	ND	ND	0.37 ± 0.00	ND	ND	2.41 ± 0.67
Histidine	26.90	139	12	0.07 ± 0.00	0.11 ± 0.01	0.06 ± 0.00	0.04 ± 0.00	0.06 ± 0.00	0.08 ± 0.02
*Isoleucine*	*14.15*	*115*	*10*	*0.67* ± 0.24	*0.83* ± 0.26	*0.53* ± 0.11	*0.36* ± 0.08	*0.62* ± 0.09	*0.39* ± 0.11
Leucine	14.10	144	6	0.40 ± 0.00	0.50 ± 0.07	0.48 ± 0.11	0.73 ± 0.17	0.40 ± 0.08	0.66 ± 0.12
Lysine	26.35	142	17	0.21 ± 0.05	0.38 ± 0.11	0.26 ± 0.02	0.34 ± 0.01	0.21 ± 0.03	0.21 ± 0.05
Methionine	18.10	147	14	0.04 ± 0.00	0.06 ± 0.00	0.04 ± 0.00	0.05 ± 0.00	0.04 ± 0.00	ND
*N*-Acetylglutamic acid	19.28	116	13	0.05 ± 0.01	0.08 ± 0.00	0.04 ± 0.01	0.08 ± 0.02	0.06 ± 0.00	0.03 ± 0.00
Ornithine	24.85	128	19	0.11 ± 0.01	0.14 ± 0.01	0.07 ± 0.00	0.19 ± 0.06	0.05 ± 0.00	ND
Phenylalanine	20.05	162	14	0.24 ± 0.04	0.37 ± 0.07	0.22 ± 0.00	0.34 ± 0.06	0.28 ± 0.02	0.46 ± 0.07
*Proline*	*15.15*	*128*	*6*	*7.31* ± 1.31	*9.26* ± 1.67	*6.81* ± 0.66	*7.55* ± 0.98	*8.74* ± 1.11	*12.35* ± 1.09
Putrescine	21.80	115	15	0.04 ± 0.00	ND	ND	0.15 ± 0.00	0.14 ± 0.05	0.07 ± 0.01
Pyroglutamic acid	16.55	84	13	ND	0.66 ± 0.11	ND	ND	ND	1.85 ± 0.87
Serine	17.45	100	14	0.05 ± 0.00	0.07 ± 0.01	0.03 ± 0.00	0.04 ± 0.00	0.05 ± 0.01	0.08 ± 0.02
Threonine	15.75	115	8	0.27 ± 0.11	0.31 ± 0.04	0.18 ± 0.02	0.13 ± 0.05	0.22 ± 0.01	0.23 ± 0.03
*Tyrosine*	*28.75*	*236*	*11*	*0.16* ± 0.00	*0.24* ± 0.05	*0.12* ± 0.00	*0.16* ± 0.00	*0.17* ± 0.04	*0.41* ± 0.09
Valine	12.85	130	10	1.16 ± 0.28	1.40 ± 0.11	0.93 ± 0.08	1.12 ± 0.04	1.09 ± 0.03	1.82 ± 0.55
**Tripeptide (1):**									
Glutathione	19.00	142	8	0.03 ± 0.00	0.05 ± 0.00	0.02 ± 0.00	ND	0.01 ± 0.00	ND
**Carboxylic acids (26):**									
3-hydroxybenzoic acid	17.01	135	16	0.02 ± 0.00	0.04 ± 0.00	0.03 ± 0.00	0.03 ± 0.00	0.02 ± 0.00	0.12 ± 0.02
4-hydroxycinnamic acid	23.22	161	16	ND	0.03 ± 0.00	0.07 ± 0.01	ND	ND	0.02 ± 0.00
2-isopropylmalic acid	12.87	145	13	0.04 ± 0.00	0.10 ± 0.03	0.07 ± 0.02	0.08 ± 0.03	0.09 ± 0.02	0.10 ± 0.02
4-hydroxyphenylacetic acid	18.89	121	23	0.05 ± 0.01	0.02 ± 0.00	0.14 ± 0.04	ND	0.26 ± 0.06	0.47 ± 0.09
2-oxoglutaric acid	13.85	115	15	0.14 ± 0.01	ND	0.13 ± 0.00	ND	ND	0.32 ± 0.l2
2-oxovaleric acid	7.17	71	24	ND	0.03 ± 0.00	0.02 ± 0.00	0.02 ± 0.00	0.03 ± 0.00	0.05 ± 0.01
Benzoic acid	9.70	105	8	0.01 ± 0.00	0.02 ± 0.00	0.02 ± 0.00	0.02 ± 0.00	0.02 ± 0.00	0.03 ± 0.01
cis-Aconitic acid	9.70	105	12	0.07 ± 0.02	0.03 ± 0.00	0.10 ± 0.01	ND	0.16 ± 0.03	1.76 ± 0.55
*Citric acid*	*15.55*	*153*	*9*	*0.20* ± 0.05	*0.45* ± 0.02	*0.26* ± 0.03	*0.15* ± 0.01	*0.25* ± 0.06	*1.94* ± 0.78
Citraconic acid	16.45	143	19	0.02 ± 0.00	0.02 ± 0.00	0.02 ± 0.00	0.02 ± 0.00	0.02 ± 0.00	0.16 ± 0.06
Citramalic acid	10.20	127	10	0.19 ± 0.05	0.11 ± 0.02	0.14 ± 0.00	0.26 ± 0.06	0.21 ± 0.03	0.22 ± 0.05
*Fumaric acid*	*10.75*	*117*	*13*	*0.94* ± 0.67	*1.03* ± 0.09	*0.61* ± 0.05	*0.44* ± 0.02	*0.98* ± 0.08	*2.68* ± 0.55
Glutaric acid	9.25	113	13	0.02 ± 0.00	0.02 ± 0.00	0.02 ± 0.00	0.03 ± 0.01	0.02 ± 0.00	0.05 ± 0.01
Glyceric acid	11.75	119	16	ND	ND	ND	0.05 ± 0.02	0.02 ± 0.00	ND
Glyoxalic acid	11.25	75	14	0.01 ± 0.00	0.01 ± 0.00	0.00 ± 0.00	ND	0.01 ± 0.00	0.01 ± 0.00
Isocitric acid	21.05	129	14	0.07 ± 0.01	0.12 ± 0.03	0.07 ± 0.01	0.06 ± 0.00	0.10 ± 0.02	0.18 ± 0.03
Itaconic acid	10.22	127	19	0.05 ± 0.00	0.16 ± 0.04	0.10 ± 0.01	0.07 ± 0.03	0.09 ± 0.02	1.14 ± 0.06
*Lactic acid*	*9.28*	*103*	*9*	*3.33* ± 0.85	*1.65* ± 0.12	*1.98* ± 0.50	*2.22* ± 0.43	*2.61* ± 0.65	*4.33* ± 1.11
Levulinic acid	8.75	99	8	0.45 ± 0.04	0.68 ± 0.08	0.47 ± 0.05	0.04 ± 0.00	0.51 ± 013	0.33 ± 0.05
*Malic acid*	*11.45*	*103*	*8*	*0.25* ± 0.07	*0.41* ± 0.05	*0.21* ± 0.11	*0.59* ± 0.16	*0.35* ± 0.54	*0.78* ± 0.54
Malonic acid	7.50	101	17	0.48 ± 0.12	0.69 ± 0.24	0.46 ± 0.07	0.40 ± 0.03	0.63 ± 0.08	1.00 ± 0.04
Oxaloacetic acid	9.73	101	32	0.13 ± 0.03	0.18 ± 0.02	0.13 ± 0.04	0.10 ± 0.01	0.13 ± 0.03	0.18 ± 0.08
*p*-Coumaric acid	10.52	164	15	0.11 ± 0.01	0.16 ± 0.03	0.07 ± 0.00	0.12 ± 0.03	0.14 ± 0.05	0.37 ± 0.11
*Succinic acid*	*9.10*	*115*	*6*	*3.80* ± 0.98	*4.86* ± 0.87	*3.53* ± 0.56	*4.03* ± 0.76	*4.51* ± 0.22	*6.87* ± 0.44
Syringic acid	24.40	211	15	0.05 ± 0.00	0.01 ± 0.00	ND	ND	0.11 ± 0.03	0.08 ± 0.02
*Tartaric acid*	*20.80*	*59*	*11*	*3.26* ± 0.78	*2.22* ± 0.55	*1.54* ± 0.34	*4.54* ± 0.13	*3.46* ± 0.24	*3.89* ± 0.11
**Fatty acids (4):**									
3-Methyl-2-oxopentanoic acid	7.69	57	14	0.01 ± 0.00	0.02 ± 0.00	0.01 ± 0.00	0.02 ± 0.00	0.01 ± 0.00	0.04 ± 0.00
4-Methyl-2-oxopentanoic acid	7.79	85	10	ND	0.02 ± 0.00	0.01 ± 0.00	ND	0.04 ± 0.01	0.06 ± 0.01
*Stearic acid*	*24.22*	*74*	*15*	*0.67* ± 0.22	*0.89* ± 0.13	*0.22* ± 0.05	*0.17* ± 0.01	*0.55* ± 0.12	*0.46* ± 0.07
10,13-Dimethyltetradecanoic acid	20.22	180	13	0.03 ± 0.01	0.03 ± 0.00	0.02 ± 0.00	0.03 ± 0.00	0.03 ± 0.01	ND
**Sugars (9):**									
d-Galactose	18.75	319	4	1.91 ± 0.76	1.79 ± 0.55	1.83 ± 0.56	2.02 ± 0.87	2.20 ± 0.26	2.94 ± 0.65
d-Glucose	19.13	319	2	1.82 ± 0.11	1.85 ± 0.45	1.89 ± 0.67	1.84 ± 0.28	2.09 ± 0.45	1.85 ± 0.16
d-Fructose	18.26	103	1	1.94 ± 0.65	1.83 ± 0.23	1.96 ± 0.41	2.01 ± 0.22	2.28 ± 0.09	1.91 ± 0.41
d-Mannose	18.72	319	8	1.92 ± 0.12	1.93 ± 0.34	1.73 ± 0.25	1.75 ± 0.16	1.85 ± 0.11	1.95 ± 0.45
d-Ribose	15.54	103	3	1.94 ± 0.56	1.83 ± 0.22	1.96 ± 0.55	2.01 ± 0.17	2.27 ± 0.21	1.91 ± 0.50
d-Sorbose	18.17	103	1	0.02 ± 0.00	0.01 ± 0.00	0.02 ± 0.00	0.02 ± 0.00	0.05 ± 0.02	0.01 ± 0.00
d-Xylose	13.98	103	4	0.02 ± 0.00	0.01 ± 0.00	0.02 ± 0.00	0.02 ± 0.00	0.05 ± 0.01	0.01 ± 0.00
Trehalose	32.40	361	6	0.01 ± 0.00	0.01 ± 0.00	0.01 ± 0.00	0.01 ± 0.00	0.02 ± 0.00	0.01 ± 0.00
Sucrose	32.76	361	4	ND	ND	ND	ND	0.02 ±	ND
**Sugar alcohols (4):**									
*d-mannitol*	*18.91*	*319*	*5*	*0.06* ± 0.02	*0.05* ± 0.01	*0.03* ± 0.01	*0.27* ± 0.06	*1.92* ± 0.65	*0.15* ± 0.02
Glycerol	10.43	147	4	0.76 ± 0.03	0.80 ± 0.09	0.97 ± 0.06	1.19 ± 0.16	0.81 ± 0.04	0.72 ± 0.05
Meso-erythritol	13.61	217	6	0.03 ± 0.00	0.03 ± 0.00	0.04 ± 0.00	0.04 ± 0.01	0.07 ± 0.01	0.03 ± 0.00
Meso-inositol	22.27	305	6	0.08 ± 0.02	0.06 ± 0.01	0.09 ± 0.02	0.09 ± 0.02	0.20 ± 0.05	0.08 ± 0.03
**Vitamin and derivative (2):**									
Nicotinic acid	10.82	137	14	0.01 ± 0.00	0.01 ± 0.00	0.01 ± 0.00	0.01 ± 0.00	0.01 ± 0.00	0.02 ± 0.00
Nicotinamide	6.04	56	20	ND	0.02 ± 0.00	0.01 ± 0.00	ND	ND	0.02 ± 0.00
**Others (2):**									
5-hydroxymethyl-2-furaldehyde	14.25	168	3	0.02 ± 0.00	ND	ND	0.12 ± 0.03	ND	ND
*Phosphate*	*11.72*	*299*	*12*	*0.15* ± 0.04	*0.13* ± 0.02	*0.15* ± 0.03	*0.28* ± 0.06	*0.50* ± 0.08	*0.15* ± 0.02

Statistically significant (*p*-value < 0.01) metabolites are shown in italics. Here, RSD = Relative standard deviation, RT = Retention time, Avg = Average, ND = Not detected, D = Delmaine^TM^, L = Lupi^TM^, M1 = Mazetti^TM^ one leaf, M4 = Mazetti^TM^ four leaves, P = Pam’s^TM^ and T = Tastemaker^TM^.

**Table 2:** Relative abundance of volatile metabolites in commercial balsamic vinegars.

Metabolite	RT (min)	*m*/*z*	Ag RSD (%), *n* = 6	Relative Abundance in Vinegar Samples					
				D	M1	L	T	M4	P
**Volatile acids (14):**									
2-Ethylhexanoic acid	9.18	88	6	ND	ND	ND	0.19	ND	ND
2-Methybutyric acid	6.59	74	7	0.23 ± 0.04	0.16 ± 0.02	0.26 ± 0.05	0.29 ± 0.08	0.24 ± 0.02	0.23 ± 0.04
4-Methyl-2-pentenoic acid	7.59	60	8	0.03 ± 0.00	ND	0.04 ± 0.01	0.05 ± 0.01	ND	0.03 ± 0.00
Acetic acid	4.43	43	4	2.26 ± 0.56	2.25 ± 0.76	2.20 ± 0.12	2.33 ± 0.09	2.20 ± 0.15	2.26 ± 0.23
*Benzeneacetic acid*	*11.13*	*91*	*7*	*0.27* ± 0.12	*0.06* ± 0.01	*0.31* ± 0.09	*0.74* ± 0.17	*0.42* ± 0.12	*0.52* ± 0.23
*Butyric acid*	*4.46*	*60*	*7*	*0.58* ± 0.11	*0.54* ± 0.16	*0.40* ± 0.09	*0.09* ± 0.02	*0.05* ± 0.00	*0.17* ± 0.04
Carbolic acid	8.56	94	9	0.05 ± 0.01	0.04 ± 0.00	0.01 ± 0.00	0.10 ± 0.02	ND	0.03 ± 0.00
*Furoic acid*	*9.75*	*112*	*8*	*0.41* ± 0.18	*0.41* ± 0.11	*0.38* ± 0.09	*0.23* ± 0.04	*0.13* ± 0.03	*0.21* ± 0.07
Hexanoic acid	8.00	60	5	0.33 ± 0.11	0.27 ± 0.06	0.24 ± 0.03	0.17 ± 0.05	0.23 ± 0.08	0.44 ± 0.11
*Isobutyric acid*	*5.44*	*73*	*6*	*1.04* ± 0.34	*0.45* ± 0.11	*1.04* ± 0.30	*0.84* ± 0.13	*0.02* ± 0.00	*0.74* ± 0.21
*Isovaleric acid*	*6.98*	*60*	*4*	*2.15* ± 0.67	*1.99* ± 0.44	*3.09* ± 0.81	*5.31* ± 0.96	*0.11* ± 0.01	*3.46* ± 0.54
Octanoic acid	10.13	73	8	0.25 ± 0.04	0.34 ± 0.02	0.19 ± 0.01	ND	0.22 ± 0.03	0.18 ± 0.03
*Propionic acid*	*5.28*	*74*	*4*	*0.84* ± 0.55	*0.46* ± 0.10	*0.54* ± 0.18	*0.18* ± 0.05	*0.25* ± 0.05	*0.14* ± 0.04
Valeric acid	7.44	60	6	0.23 ± 0.04	0.08 ± 0.01	0.05 ± 0.00	ND	ND	0.37 ± 0.06
**Esters (12):**									
2-Carboxymethyl-3-n-hexylmaleic acid anhydride	12.47	126	7	0.22 ± 0.05	0.21 ± 0.02	0.31 ± 0.05	0.12 ± 0.01	0.15 ± 0.03	0.31 ± 0.08
*(−)-Ethyl L-Lactate*	*5.65*	*45*	*7*	*0.08* ± 0.02	*0.39* ± 0.14	*0.12* ± 0.05	*0.10* ± 0.01	*0.04* ± 0.00	*0.12* ± 0.02
2-Methyl-1-butyl acetate	5.37	70	6	0.10 ± 0.02	0.17 ± 0.03	0.07 ± 0.00	0.08 ± 0.02	0.01 ± 0.00	0.06 ± 0.01
1,3-Propylene diacetate	8.76	43	9	0.17 ± 0.05	0.05 ± 0.00	0.05 ± 0.00	0.06 ± 0.01	0.16 ± 0.05	0.03 ± 0.00
*Diethyl succinate*	*9.59*	*129*	*5*	*0.41* ± 0.17	*0.71* ± 0.26	*0.87* ± 0.21	*0.15* ± 0.05	*0.03* ± 0.00	*0.14* ± 0.03
Ethylisovalerate	4.91	40	9	0.01 ± 0.00	ND	0.01 ± 0.00	0.01 ± 0.00	0.02 ± 0.00	0.01 ± 0.00
Ethyl hydrogen succinate	10.47	101	4	2.10 ± 0.23	1.89 ± 0.55	2.28 ± 0.38	2.32 ± 0.43	1.26 ± 0.17	1.17 ± 0.17
*Isoamyl acetate*	*5.34*	*43*	*6*	*0.83* ± 0.34	*1.65* ± 0.33	*0.61* ± 0.09	*0.09* ± 0.00	*0.16* ± 0.03	*0.42* ± 0.11
Methyl 2-furoate	4.98	95	8	0.03 ± 0.00	0.02 ± 0.00	0.01 ± 0.00	0.01 ± 0.00	0.04 ± 0.01	0.01 ± 0.00
Methylsuccinic anhydride	9.52	42	4	0.04 ± 0.00	0.04 ± 0.00	0.03 ± 0.01	ND	0.03 ± 0.00	ND
*Phenethyl acetate*	*10.29*	*104*	*8*	*0.44* ± 0.17	*0.44* ± 0.11	*0.55* ± 0.08	*0.24* ± 0.04	*0.12* ± 0.02	*0.21* ± 0.06
*p*-Hydroxycinnamic acid, ethyl ester	15.98	147	7	0.01 ± 0.00	0.04 ± 0.00	0.16 ± 0.05	0.06 ± 0.00	0.14 ± 0.03	0.05 ± 0.00
**Higher alcohols (9):**									
2,3-butanediol	5.76	45	7	0.42 ± 0.08	0.41 ± 0.11	0.50 ± 0.18	0.43 ± 0.09	0.32 ± 0.07	0.39 ± 0.10
*1,2,3-Benzenetriol*	*13.40*	*126*	*5*	*0.15* ± 0.02	*0.21* ± 0.04	*0.73* ± 0.21	*0.13* ± 0.06	*0.39* ± 0.11	*0.22* ± 0.06
1,4-Benzenediol	12.34	81	8	0.19 ± 0.05	0.17 ± 0.03	0.10 ± 0.01	0.13 ± 0.02	ND	0.17 ± 0.11
*2-Methyl-1-butanol*	*4.05*	*57*	*3*	*1.40* ± 0.45	*1.78* ± 0.25	*1.45* ± 0.11	*0.73* ± 0.08	*0.16* ± 0.04	*0.99* ± 0.17
*2-Phenylethanol*	*8.81*	*107*	*4*	*4.10* ± 1.08	*5.55* ± 1.16	*4.38* ± 0.98	*2.56* ± 0.45	*1.81* ± 0.76	*2.39* ± 0.54
Benzyl alcohol	8.45	79	5	0.18 ± 0.06	0.22 ± 0.04	0.13 ± 0.06	0.14 ± 0.03	0.22 ± 0.06	0.11 ± 0.02
*Isoamyl alcohol*	*5.32*	*70*	*3*	*2.70* ± 0.76	*5.67* ± 0.76	*2.02* ± 0.32	*1.01* ± 0.32	*1.02* ± 0.06	*2.26* ± 0.43
Methionol	7.79	106	9	0.04 ± 0.00	0.18 ± 0.04	0.02 ± 0.00	ND	ND	ND
*Tyrosol*	*13.43*	*107*	*3*	*0.73* ± 0.16	*0.43* ± 0.08	*0.48* ± 0.09	*0.53* ± 0.08	*0.05* ± 0.00	*0.26* ± 0.07
**Aldehydes and ketones (6):**									
1,3-Diacetoxypropane *	7.75	61	8	0.41 ± 0.06	0.16 ± 0.03	0.19 ± 0.04	0.06 ± 0.00	0.51 ± 0.12	0.07 ± 0.00
4,4-Diethyl-3-methylene-2-oxetanone	15.09	14	25	0.08 ± 0.00	0.04 ± 0.00	0.10 ± 0.02	0.04 ± 0.00	0.15 ± 0.04	0.08 ± 0.01
3-Methyl-1,2-cyclopentanedione	8.21	112	13	0.02 ± 0.00	ND	0.02 ± 0.00	0.05 ± 0.00	ND	0.02 ± 0.00
5-Hydrxoymethylfurfural	11.34	97	10	0.46 ± 0.12	0.34 ± 0.04	0.43 ± 0.08	0.23 ± 0.04	4.15 ± 0.78	0.41 ± 0.23
5-Methyl furfural	7.41	110	5	0.16 ± 0.03	0.18 ± 0.02	0.24 ± 0.08	0.01 ± 0.00	0.06 ± 0.00	0.14 ± 0.03
Acetoin	7.17	43		0.26 ± 0.06	0.14 ± 0.04	0.15 ± 0.02	0.22 ± 0.06	0.36 ± 0.10	0.16 ± 0.02
Butyrolactone	4.65	42	6	0.03 ± 0.00	0.04 ± 0.00	0.03 ± 0.00	0.03 ± 0.00	0.01 ± 0.00	0.01 ± 0.00
**Others (6):**									
2-Acetyl-1-pyrroline *	8.87	66	12	ND	ND	ND	0.01 ± 0.00	0.05 ± 0.01	0.03 ± 0.00
4-Anisidine *	8.37	45	4	ND	ND	ND	ND	0.21 ± 0.05	0.18 ± 0.04
2-Methylpyrazine *	4.96	94	2	ND	ND	ND	0.08 ± 0.01	0.01 ± 0.00	0.05 ± 0.00
Acetin *	10.00	43	8	ND	ND	0.03 ± 0.00	0.12 ± 0.03	0.22 ± 0.06	ND
Coumaran	10.97	120	4	0.89	0.64 ± 0.11	0.81 ± 0.16	0.68 ± 0.09	0.45 ± 0.05	0.90 ± 0.02
N-acetyl tyramine	13.85	107	10	0.18 ± 0.04	0.18 ± 0.07	0.13 ± 0.05	0.16 ± 0.02	0.03 ± 0.00	0.09 ± 0.02

* Represents the first detection of identification of these metabolites in balsamic vinegars. Volatile metabolites with *p*-value < 0.01 are shown in italics. RT = Retention time; Avg = Average; RSD = Residual Standard Deviation; ND = not detected, D = Delmaine^TM^, L = Lupi^TM^, M1 = Mazetti^TM^ one leaf, M4 = Mazetti^TM^ four leaves, P = Pam’s^TM^ and T = Tastemaker^TM^.

**Table 3:** The commercial balsamic vinegars analyzed, and their origins.

Brand Name	Type of Vinegar	Code	Origin	Bottling Site
Mazzetti^TM^	L’originale balsamic vinegar of Modena, four leaves	M4	Modena, Italy	Pertini 440-41032, Cavezzo
Mazzetti^TM^	L’originale aceto balsamic vinegar of Modena IGP, one leaf	M1	Modena, Italy	Pertini 440-41032, Cavezzo
Lupi^TM^	Aceo balsamic vinegar of Modena IGP	L	Modena, Italy	Montanara 22/24 41051
Pam’s^TM^	Balsamic vinegar of Modena	P	Modena, Italy	n°CSQA 216311
Delmaine^TM^	Balsamic vinegar of Modena IGP	D	Modena, Italy	n°CSQA 216311
Tastemaker^TM^	Aged balsamic vinegar of Modena, premium	T	New Zealand	Merton Road, Fernside

## Abbreviations

The following abbreviations are used in this manuscript:

GC-MS	Gas chromatography coupled to mass spectrometry
HCA	Hierarchical Cluster Analysis
MCF	Methylchloroformate
NMR	Nuclear magnetic resonance
RSD	Residual standard deviation
TVB	Traditional balsamic vinegars
TMS	Trimethylsilyl
